# Supplementary material for: Preparation and Characterization of Soft-Hard Block Copolymer of 3,4-IP-b-s-1,2-PBD Using a Robust Iron-Based Catalyst System
Source: Polymers (Basel). 2024 Apr 21;16(8):1172. doi: 10.3390/polym16081172 (PMC11053549; doi:10.3390/polym16081172)
Supplement: Supplementary file 1 [file polymers-16-01172-s001.zip › polymers-2951632-supplementary.pdf]

Supporting Information

Preparation and Characterization of Soft-Hard Block Copolymer of 3,4-IP-b-s-1,2-PBD Using a Robust Iron-Based Catalyst System

Table of contents

Figure S1. The additive employed in iron-catalyzed IP polymerization............**S3**

Figure S2. Comparison of polymerization behavior of three commercially available donors for IP polymerization...**S3**

Table S1. Variation of temperature and time on IP polymerization...........................**S3**

Table S2. Mechanical properties of 3,4-PI, *s*-1,2-PBD and 3,4-PI-*b*-*s*-1,2-PBD with various soft to hard segment lengths..**S4**

Table S3. Tan δ values of 3,4-PI, *s*-1,2-PBD and representative 3,4-PI-*b*-*s*-1,2-PBD (Run 22)...**S4**

Figure S3. GPC profiles of polymers obtained from two-step polymerization of IP...**S4**

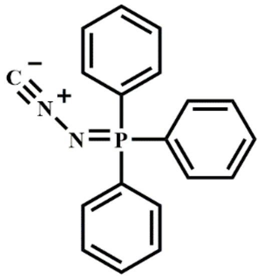

(isocyanoimino) triphenylphosphorane (**IITP**)

Figure S1. The additive employed in iron-catalyzed IP polymerization.

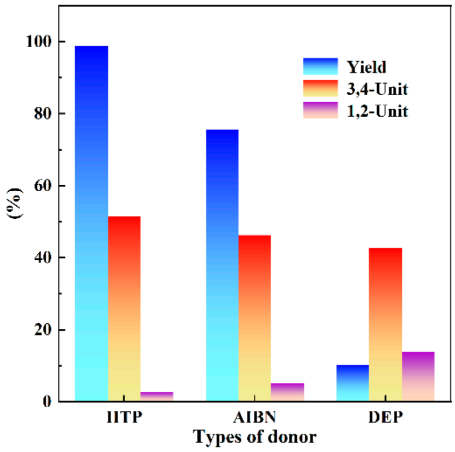

Figure S2. Comparison of polymerization behavior of three commercially available donors for IP polymerization. Polymerization conditions: in hexane at 50 °C for 4 h, [IP]=2.3 mol L^{-1}, [IP]/[Fe]=7000, [P]/[Fe]=3, and [Al]/[Fe]=20 (mol/mol).

Table S1. Variation of temperature and time on IP polymerization [a]

Run	Temp. (°C)	Time (h)	Yield (%)	Activity [b]	M_n [c] ($\times 10^4$)	PDI [c]	Microstructure [d] (%) 3,4	1,2	1,4	T_g [e] (°C)
1	0	24	7.9	3.4	29.2	1.64	54.6	2.2	43.2	−15.8
2	10	2	4.0	20.4	22.6	1.90	54.1	2.5	43.5	−14.5
3	20	2	13.9	70.9	45.6	1.77	52.7	2.6	44.7	−16.3
4	30	2	81.5	415.7	63.7	1.77	52.7	2.5	44.9	−16.3
5	50	0.6	79.9	1358.3	76.6	1.95	49.7	3.7	46.7	−16.8
6	50	2	96.7	493.2	90.4	1.93	51.3	3.3	45.4	−16.8
7	70	0.15	79.5	5406.0	81.6	1.88	49.5	3.8	46.7	−17.5
8	70	0.25	92.7	3782.2	74.6	1.92	49.8	3.5	46.7	−17.2

[a] Polymerization conditions: in hexane, [IP]=2.3 mol L^{-1}, [IP]/[Fe]=15000, [P]/[Fe]=3, and [Al]/[Fe]=20 (mol/mol); [b] Activity in units of g $mol_{(Fe)}^{-1}$ h^{-1}; [c] Determined by GPC (using polystyrene as calibration); [d] Determined by NMR; [e] Determined by DSC.

Table S2. Mechanical properties of 3,4-PI, *s*-1,2-PBD and 3,4-PI-*b*-*s*-1,2-PBD with various soft to hard segment lengths.

Samples	Tensile strength (MPa)	Elongation at break (%)
Run 19	0.58±0.09	586±39
Run 20	4.75±0.12	579±14
Run 21	5.83±0.26	492±31
Run 22	7.46±0.24	434±27
Run 23	8.66±0.12	400±19
Run 24	13.36±0.13	348±25

Table S3. Tan δ values of 3,4-PI, *s*-1,2-PBD and representative 3,4-PI-*b*-*s*-1,2-PBD (Run 22).

Samples	Tan δ at 0°C	Tan δ at 60°C
Run 19	2.278	0.032
Run 20	1.635	0.048
Run 24	0.211	0.088

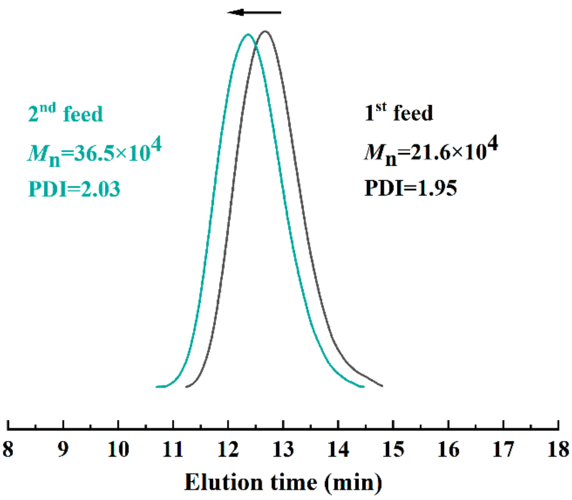

Figure S3. GPC profiles of polymers obtained from two-step polymerization of IP. Polymerization conditions: in hexane, $[IP]_0$=0.5 mol L^{-1}, $[IP]_0$/[Fe]=600, [P]/[Fe]=3, [Al]/[Fe]=20, and $[IP]_{total}$/[Fe]=1200 (mol/mol).
